# Biopsy Proven Focal Osteolysis in a Stainless-Steel Limb-Lengthening Device: A Report of Three Cases

**DOI:** 10.5435/JAAOSGlobal-D-21-00101

**Published:** 2021-10-13

**Authors:** Oliver C. Sax, Diana W. Molavi, John E. Herzenberg, Shawn C. Standard, Philip K. McClure

**Affiliations:** From the International Center for Limb Lengthening, Rubin Institute for Advanced Orthopedics, Sinai Hospital of Baltimore, Baltimore, MD.

## Abstract

Three pediatric patients presented with histologically confirmed osteolysis after limb lengthening with a magnetic, telescoping, stainless-steel device. The first patient's findings were discovered radiographically before routine removal of the device. In all cases, intraoperative histologic specimens taken from around the modular junction demonstrated particle-laden macrophages with suspicion for metal debris. Silicone debris was also identified.

We found definitive osteolysis secondary to metal at the modular junction of three stainless-steel lengthening implants. This process is not well-understood in the setting of limb lengthening and should be examined further.

Intramedullary limb lengthening remains the standard treatment modality for patients with limb-length discrepancies. Advancements since Ilizarov's external fixation device^1,2^ have nearly eliminated complications such as superficial infection, pain, and joint stiffness.^3,4,5,6^ An intramedullary titanium device (Precice, NuVasive) was introduced in 2011 and quickly garnered attraction because of its noninvasive and practical lengthening course.^7^ The Precice Stryde Nail system (NuVasive) was approved in 2018 with promises of earlier weight-bearing and potentially earlier consolidation.^8^ As part of the Precice Stryde system, both intramedullary lengthening nails and lengthening plates are available. This system uses a magnetic, telescoping stainless-steel device capable of distraction under the control of an external remote controller. The gasket ring situated at the modular junction is a composite of ethylene-propylene-diene-monomer rubber lined by silicone. Precice Stryde–related complications have been limited to hardware failure, and recent concerns have emerged regarding local bone changes at the implant junction and hardware failure on removal. Previous literature regarding osteolysis in the setting of metal implants is largely in reference to metal-on-metal total hip arthroplasty (THA), but this process is poorly understood in the setting of limb-lengthening devices.

Periprosthetic osteolysis is a macrophage-driven destructive process that can lead to implant failure because of component loosening. The presence of wear particles, such as those seen in the setting of hip arthroplasty secondary to micromotion at the modular junction, causes an inflammatory reaction leading to bone resorption.^9,10,11,12,13,14^ Silicone is another recognized culprit of particulate wear and reaction, especially in hand arthroplasty.^15,16^ To date, only a single study described a periosteal reaction and osteolysis in the setting of intramedullary nails.^17^ The authors subsequently abandoned the use of the device. Recently, Johnson et al^18^ detailed a case report of a Precice Stryde nail dissociation during routine intraoperative removal. They did not report any soft-tissue reaction or bony changes as possible causes for the intraoperative complication. Despite some reported complications associated with intramedullary lengthening nails^19^ and reports of wear occurring at the modular junction,^20^ osteolysis has yet to be described in association with these intramedullary lengthening devices.

We present three cases of periosteal reactions and osteolysis after intramedullary nail or lengthening plate implantation, with associated histologic evidence of wear particles. Institutional Review Board exemption was provided in this retrospective case report. Patient-specific details and radiographic images have been deidentified according to patient confidentiality standards.

## Case Report

### Case 1

**Figure 1:**
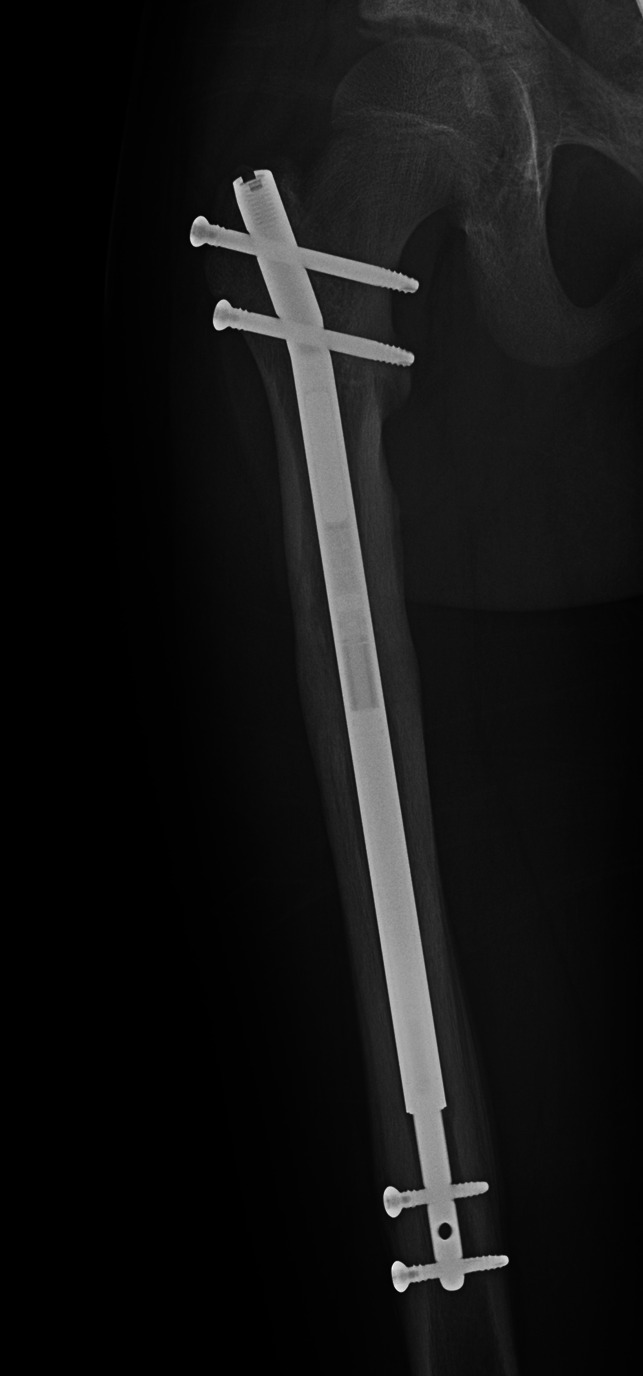
AP radiograph demonstrating a right femur approximately 5 months postimplantation of a stainless-steel intramedullary lengthening nail.

**Figure 2:**
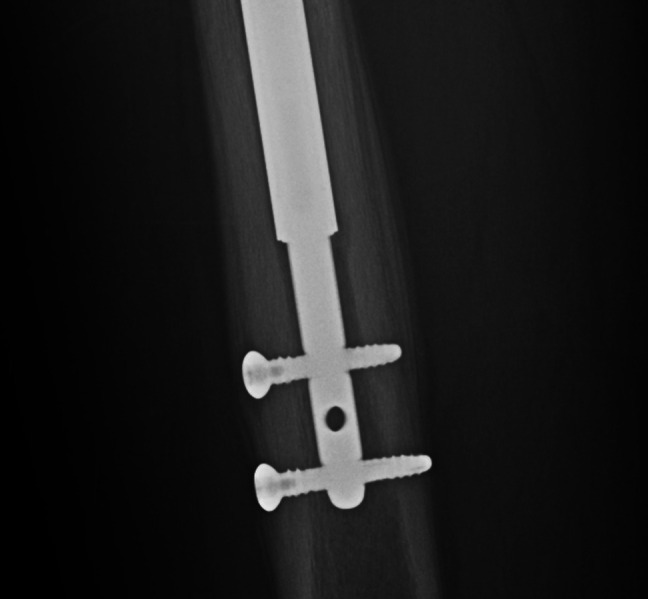
AP radiograph (with 135% magnification) of a right femur demonstrating that a punched-out appearance resembling osteolysis is appreciated medial to the modular junction. Onion skinning resembling periosteal reaction is appreciated along the medial femoral shaft adjacent to the modular junction and around both ends of the distal locking screws.

**Figure 3:**
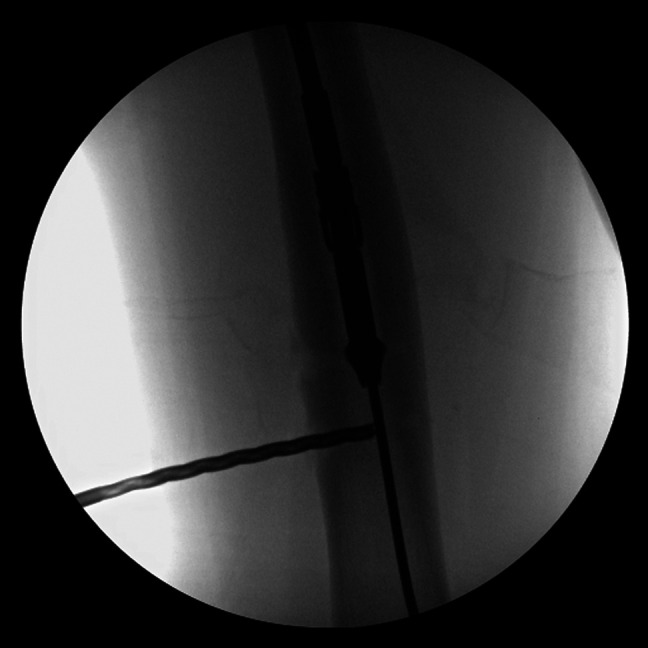
Intraoperative fluoroscopy image demonstrating the Reamer Irrigator Aspirator system reamer placement in the femoral canal at the level of the previously removed modular junction of a stainless-steel intramedullary lengthening nail.

**Figure 4:**
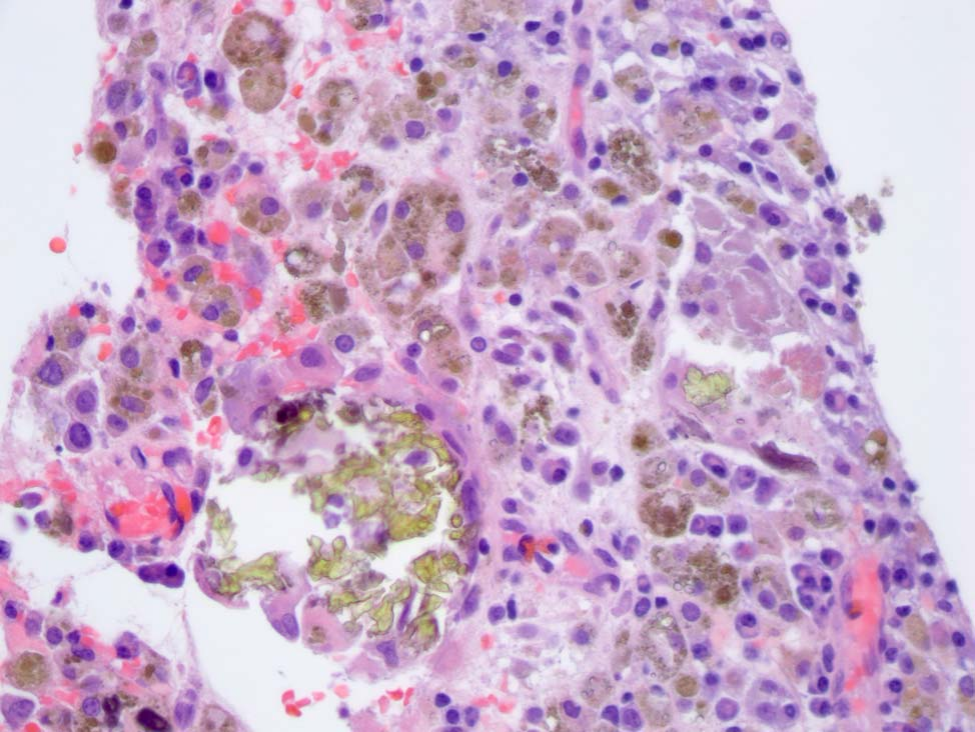
Image from reamings on case 1 showing macrophages full of granular dark particles and extracellular, larger greenish-gold crystalline debris particles (×400). Although the intracellular particles show morphologic overlap with hemosiderin, the larger crystalline fragments are not typical of hemosiderin deposition.

**Figure 5:**
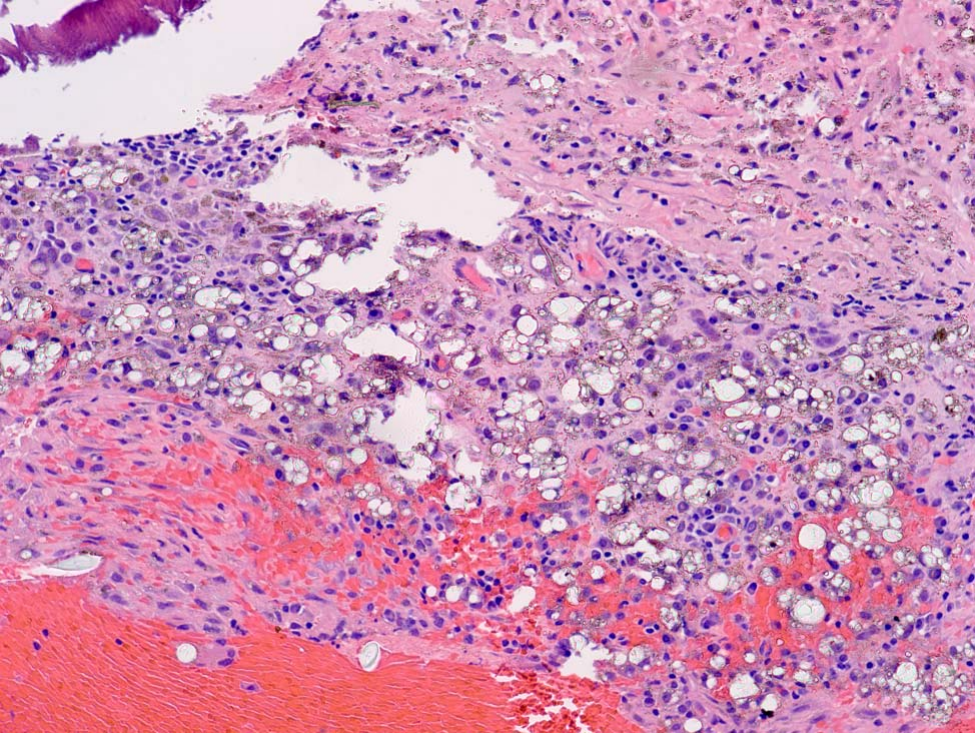
Image from reamings on case 1 showing abundant fragments of refractile, clear material, both intracellular and extracellular (×200). The material was not birefringent, consistent with silicone debris.

**Figure 6:**
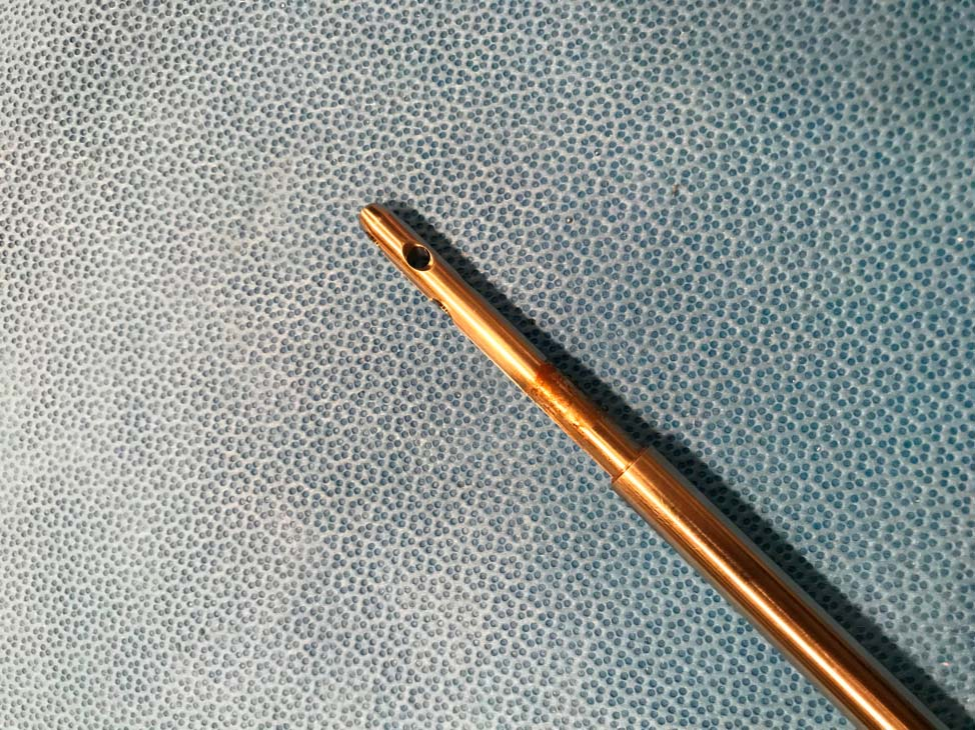
Photograph showing the explanted lengthening nail of case 1. Nail distracted to reveal corrosive changes at the male end of the modular junction.

The first patient was a 14-year-old adolescent boy with a history of posttraumatic limb-length discrepancy (LLD) of the right femur who underwent intramedullary lengthening. Preoperative LLD measured 2.1 cm, and serum chromium level measured 0.8 μg/L (normal range: 0.1 to 2.1). At the time of rod removal, radiographs demonstrated osteolysis at the modular junction of the telescopic nail (Figures 1 and 2). To determine the etiology of the osteolysis, a biopsy was done using the Reamer Irrigator Aspirator system (DePuy Synthes). The intramedullary guide rod was manipulated through the residual hole in the diaphysis from the interlocking nails. A cannulated drill bit was used to drive the guidewire and reamer into the medial cortex of the femur, which was disproportionately affected by the osteolysis (Figure 3). The reamer was pushed to the level of the lesion without activating the reamer. The irrigation/aspiration and reaming were then activated 1 cm above the lesion and deactivated 1 cm distal to the lesion. Intramedullary contents were collected in the suction trap. The remainder of the canal was then reamed and collected as a separate specimen. The biopsies were processed using routine histology procedures, including formalin fixation, xylene-based tissue processing, embedding in paraffin wax, and staining with hematoxylin and eosin. Microscopic sections showed an abundance of particulate debris, including fine brown-to-black particles taken up by macrophages and large crystalline fragments with a green-yellow color (Figure 4). These larger fragments are similar to those described in 2001 by Jones et al,^17^ which were found to contain chromium, consistent with stainless-steel components. In our patient, there was also abundant clear, refractile, nonbirefringent material consistent with silicone debris (Figure 5). Postoperative evaluation of the explanted nail demonstrated significant corrosion at the modular junction (Figure 6).

### Case 2

**Figure 7:**
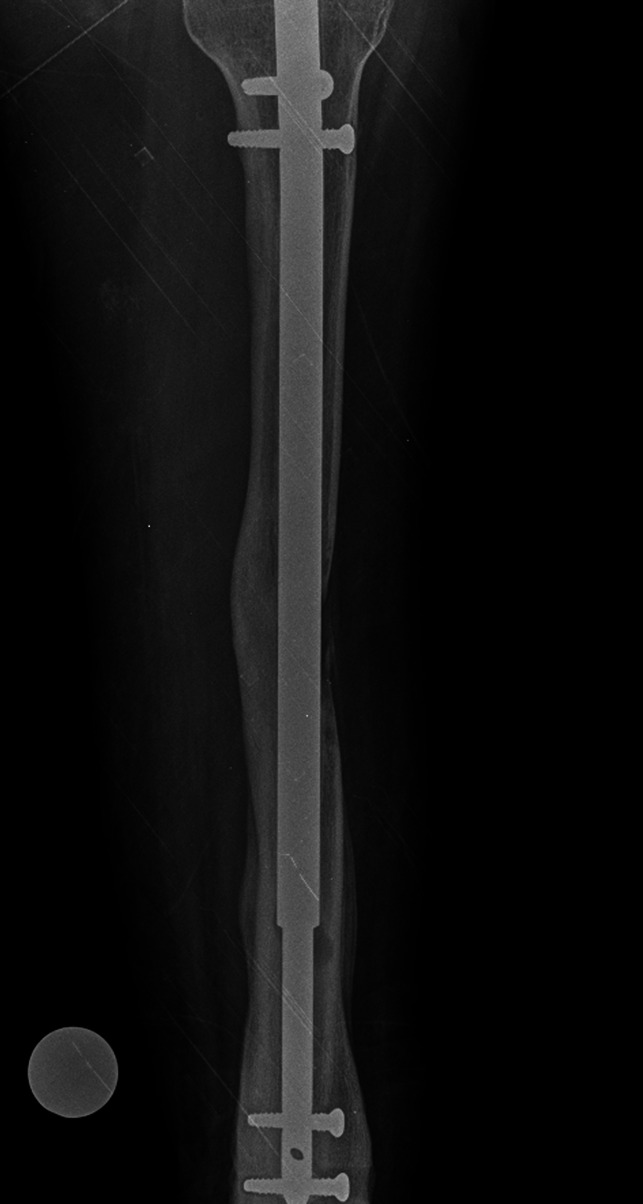
AP radiograph of a right tibia demonstrating a punched-out appearance distal and medial to the modular junction resembling osteolysis.

The second patient was a 17-year-old adolescent girl with a history of right lower extremity congenital limb deficiency who underwent intramedullary lengthening of the right tibia. Approximately 22 months after implantation, osteolysis was suspected (Figure 7), and tibial nail removal was completed with prophylactic plating given suspicion for possible osteomyelitis (later determined to be negative). Preoperative LLD measured 4.0 cm, and serum chromium level measured 1.9 μg/L. Biopsies were taken from a defect on an anterolateral defect of the tibia, and a medial bridge plate was placed. The reamings showed abundant crystalline debris and macrophages full of granular dark material; however, silicone debris was not observed.

### Case 3

**Figure 8:**
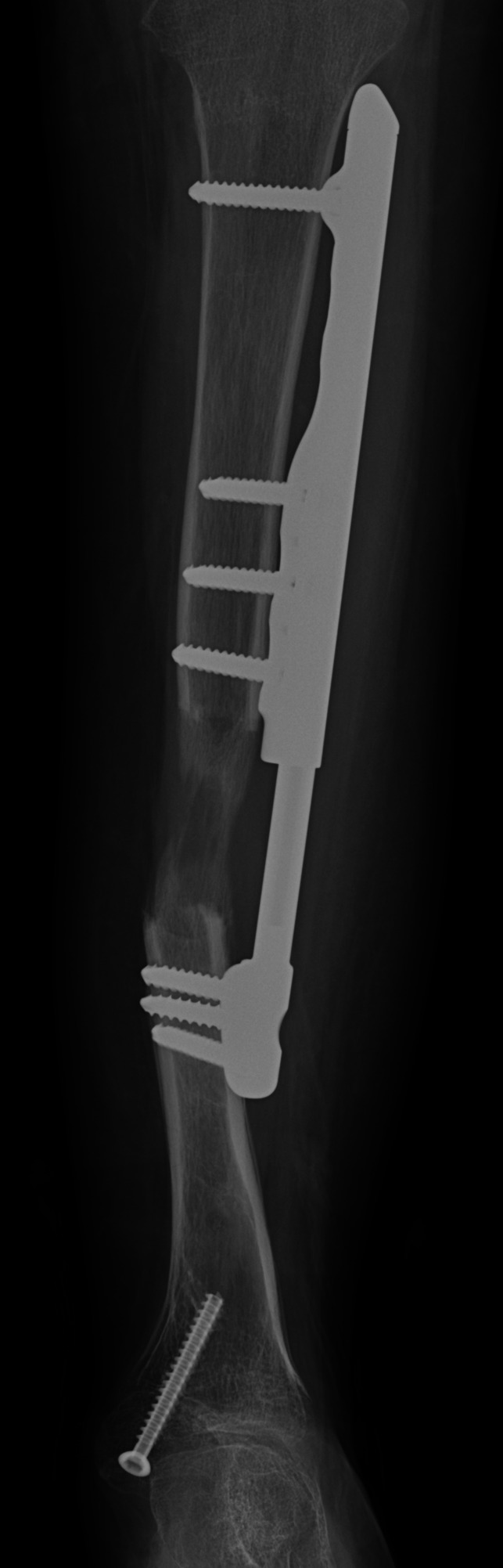
AP radiograph demonstrating a left tibia approximately 2 months after placement of an extramedullary stainless-steel limb-lengthening device. Tibial valgus deformity is appreciated.

**Figure 9:**
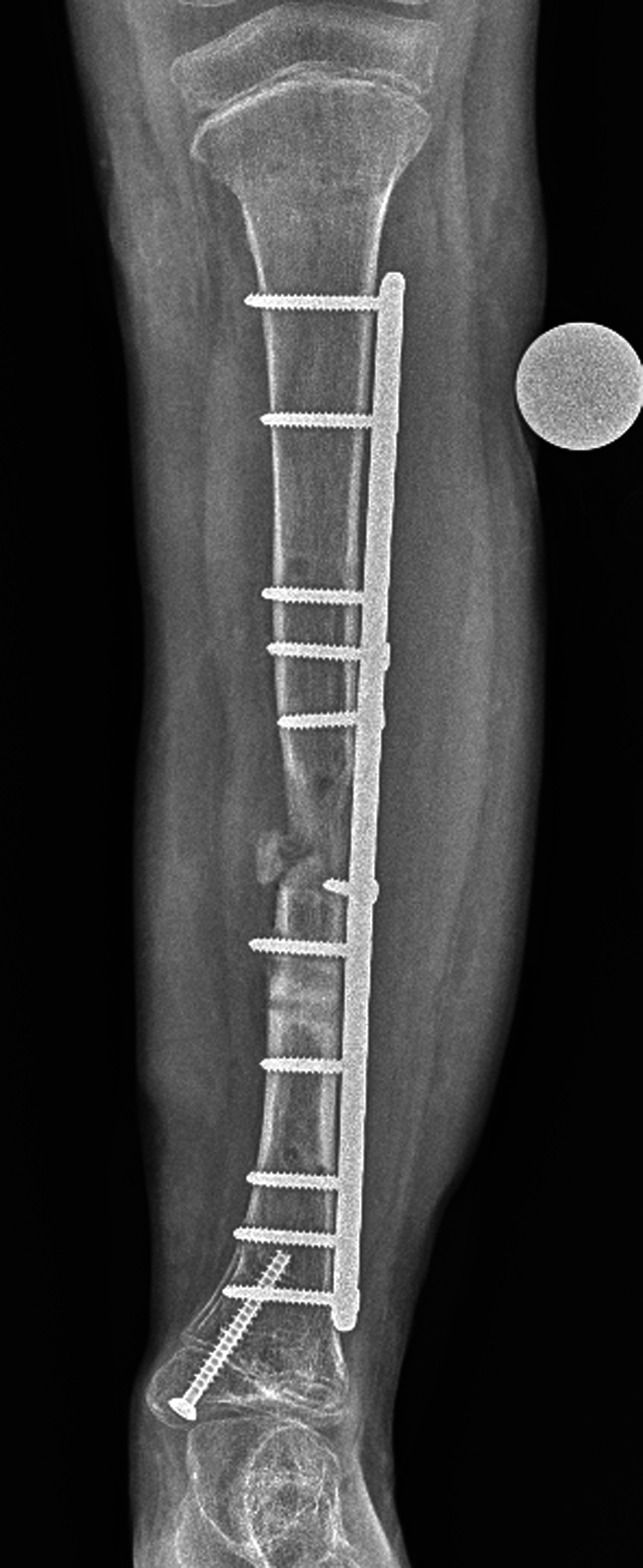
Postoperative AP radiograph demonstrating a left tibia after an external fixation-assisted plate exchange for tibial realignment.

**Figure 10:**
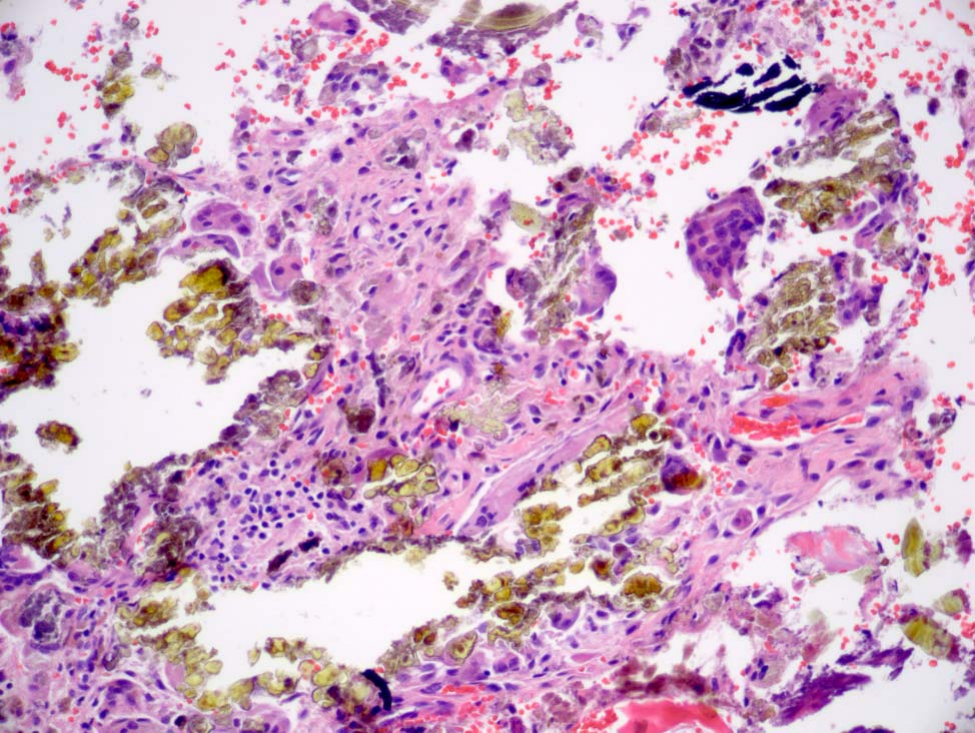
Image from case 3 showing abundant fragments of golden-brown crystalline debris and occasional fragments of black metallic material (top right; ×200). Foreign body giant cells and lymphoplasmacytic inflammation are present.

The third patient was a 10-year-old boy with a history of congenital femoral deficiency and fibular hemimelia who underwent removal of a left tibial-lengthening plate. Preoperative LLD measured 6.0 cm. Hardware failure at the distal fixation block created a tibial residual valgus/procurvatum deformity during distraction with 2.5 cm length achieved (Figure 8). Fixator-assisted plating was used to correct the deformity (Figure 9). Biopsy samples were taken from the superficial and deep surface of the plate and the underlying bone. The soft tissue adjacent to the plate in this patient showed abundant particle-laden macrophages and greenish-gold crystalline debris, similar to that seen in the first patient (Figure 10). Silicone debris was present in this patient, although considerably less than that seen around the intramedullary nail of the first patient.

## Discussion

Osteolysis is often associated with the inflammatory response to particulate debris generated by micromotion at device junctions, such as the frictional interface between weight-bearing surfaces of THA or the modular junction of a telescoping rod.^17,18,20^ A comparison between modular intramedullary nails and one-piece nails found that the periosteal reaction and osteolysis were found specifically at the junctions and were not seen in patients with a one-piece nail.^17^ However, the phenomenon does not occur in all devices and has not been previously described in conjunction with limb-lengthening nails, suggesting that some component materials may be more resistant to wear or corrosion than others. Despite implantation of over 10,000 titanium-lengthening rods globally, no reports of osteolysis exist.

This report is not without its limitations. Although macrophage-ingested metal particles and silicone were identified on histologic analysis in both patients, we cannot definitively confirm how much of association this has to the development osteolysis. Furthermore, this report describes a phenomenon in the context of a nail-lengthening and plate-lengthening device. Although their lengthening processes commence at different mechanical sites (intramedullary versus extramedullary), their function to distract bone in the coronal plane is nearly identical. These limitations should not be disqualifying given the nuanced reporting of a poorly understood process in limb lengthening.

Metal-on-metal hip arthroplasty is perhaps the most prominent example of osteolysis, occurring between two surfaces of high relative motion.^9,10,11,12,13,14^ Kitamura et al^21^ reported the natural progression to pelvic osteolysis after THA averaged 1.3 years. The intra-articular nature of the bearing surface in metal-on-metal or metal-on-polyethylene THA limits direct comparison with our series. Similarly, trunnion wear is also limited by the intra-articular nature of the surface.^11^ A modular trauma nail generated periosteal reaction at an average of 9 months, osteolysis at 13 months, and cortical thickening at 14 months.^17^ Earlier findings of osteolysis in our patients indicate the possibility of a more severe reaction. Several differences may account for this accelerated time frame. The lengthening junction in telescopic nails has more motion than a press-fit modular taper, which increases the stress on implant surfaces. In addition, distraction of the osteotomy increases the stress on the implant relative to fracture fixation. Although increased chromium levels are associated with osteolysis progression,^22^ the measured levels in the presented patients were within the normal range.

Our cases showed evidence of corrosion, metallic particles, and silicone debris, previously reported to cause osteolysis.^17,23^ Silicone debris has also been shown to create osteolysis in isolation,^15,16^ with a potential negative synergistic response biologically. The relative importance of each factor requires additional study to elucidate. We report the biopsies from patients with lysis around the junction, not indicative of a causative factor other than metal particles alone. The silicone does not contribute to third body wear because of its pliability, and no other metallic material was identified, indicating that a breakdown in the hermetically sealed components does not seem to contribute to osteolysis. This should not be interpreted as indicating that the mechanism is in fact sealed from the biologic space of the intramedullary canal; however, the particles identified are consistent with corrosion products from previous modular nails without a magnetic lengthening drive.

In conclusion, we find definitive evidence of osteolysis secondary to metal at the modular junction of three stainless-steel lengthening implants. Although this has not been previously reported, the implant remains relatively new. The relative contribution of various factors to the osteolysis requires additional study to determine whether a subset of patients are particularly susceptible. Despite these conclusions, an intramedullary titanium nail (Precice nail, NuVasive) continues to be an excellent alternative.
